# Cultural evolution in adaptive management of grassroots activism in BC, Canada

**DOI:** 10.1007/s11625-017-0512-7

**Published:** 2017-12-04

**Authors:** Karl Frost

**Affiliations:** 1 University of California, Davis, USA; 2 ICTA, Universitat Autonoma de Barcelona, Barcelona, Spain; 3 Department of Human Behavior, Ecology, and Culture, Max Planck Institute for Evolutionary Anthropology, Leipzig, Germany

**Keywords:** Cultural evolution, Grassroots activism, Sustainability, First Nations, Bonding

## Abstract

This paper demonstrates how implicit cultural evolution theory (CE) is used in adaptive management of grassroots campaigns of resistance against environmentally destructive industry and government to facilitate sustainable outcomes. For an action to be sustainable, it must be stable against political pressures. By bringing attention to the effects of social transmission—recruitment to a cause, learning across campaigns, and the transmission or cultivation of solidarity sentiments—cultural evolution presents a framework for tracking social dynamics essential for the sustainability of resistance projects. This is illustrated with examples from direct action grassroots activism in First Nations communities in northern British Columbia, Canada in the context of fights against unsustainable industrial projects. Specifically, grassroots activists work with an implicit CE theory of social transmission of values that posits that expansive, large-group organizing can get large numbers moderately committed to cause but that organizing focusing on small groups is more successful at transmitting intense commitment and adherence to First Nations norms. In the case of direct action resistance, such intense commitment is more vital than numbers for success. Further, grassroots activists have self-consciously developed institutions for the rapid transmission of policy innovations, accelerating the constructive evolution of tactics.

## Introduction

For many indigenous communities, the easy part of the sustainability question is managing environmental relationship, with millennia of accumulated traditional ecological knowledge (Turner et al. 2000). The difficult part of the sustainability question is the political one of sustaining resistance to colonial governments and corporations. Key questions include how to recruit adequate numbers to resistance, how to cultivate adequate levels of commitment and willingness to sacrifice, and how to transfer skills amongst campaigns. These questions concern social transmission of behaviors, the subject of cultural evolution theory (CE), and the theme of this special issue. Demonstrating how grassroots First Nations activists in northern BC, Canada use implicit cultural evolution reasoning, this paper focuses on a practice-based theory of social transmission of intense value commitment in the context of direct action resistance. It also documents the development of institutions for managing the constructive evolution of strategy amongst First Nations. It is “implicit” in the sense that activists consider social transmission processes and strategize accordingly, but do not make reference to the academic vocabularies of cultural evolution. Integrating social and environmental sciences, I explore the question of *political* sustainability in the context of defending traditional lifeways against industrial incursions.

Analysis is supported by established social science and informed by qualitative research, participant observation, and historical anecdotes. Unless cited otherwise, information about ongoing and recent actions is from personal participant observation and unstructured interviews and conversations with action leaders and participants, 2014–2016 (Frost 2016). While I did conduct structured interviews, less structured conversations were more fruitful in detailing the complexities of real organizing in complex communities. Many of these campaigns are legally contested. For example, while government officials have called occupations on Unist’ot’en territory ‘illegal’, the Supreme Court has ruled in support of the sovereignty of First Nations on their traditional territory, and while non-treaty First Nations may embrace court support for their causes when it comes, they reject court authority to decide anything on their territory. The often volatile legal circumstances create unique problems for reporting on specific details. There is the desire to share information in a way that helps to normalize necessary environmental and social defense actions, balanced with discretion. For this reason, I restrict myself to less sensitive, public information and widely, openly held views within activist community, holding the intention to be respectful and constructive with the information I share from personal experience and conversations.

First Nations regularly face threats to their territories and their communities from resource extraction industries. Traditional lifeways have been deliberately and violently suppressed by the Canadian government to make way for rapid and unsustainable resource extraction with profits going to white colonial industry owners (King 2012). These fights are ongoing, and grassroots activists have explored multiple strategies.

The practice-based theory of resistance explored in this paper posits that while large-scale organizing via established NGOs can quickly build large networks of support, this support is relatively ‘shallow’, with individuals having limited willingness to sacrifice. Where government can inflict severe consequences on resistors, greater commitment is necessary. Smaller groups develop tighter bonds and stronger commitment to sacred values through intimate shared experience of sacrifice. This stronger commitment allows smaller numbers to engage in high-risk tactics that in some circumstances hold off industry more effectively. This theory strongly parallels social science research, detailed below.

In adaptively managing the social transmission of solidarity, activists use an implicit CE theory. Cultural evolution is the study of social transmission of behaviors and the resulting rise, maintenance, or decline of these behaviors (Boyd and Richerson 1985). Social transmission takes many forms, including teaching or copying. Rather than a population instantly switching from one behavior to another, introduced behaviors diffuse into a population over time from individual to individual. Classical micro-economic analysis presents a commonly used counter model, treating people as rational, well-informed actors making individual choices from amongst all possible options based on well-ordered preferences. What is left out of this counter model is the flow of information and sentiment through a society, as well as how social information (observed adoption of a behavior by others) affects individual choices to adopt. Analysis gets more realistic by modeling how decisions are made amongst known options with new options revealing themselves through adoption choices of others and how these choices are sometimes not rational choices based on values, but the learning of the values themselves. The resulting diffusion patterns of novel behaviors through a population via social transmission have been empirically documented widely in the literature on diffusion of innovation (Rogers 2003). This process of social transmission of behaviors is modeled similarly with different vocabulary in different fields, including diffusion of innovation, CE, and evolutionary economics. While there are variations in approaches, the models are often interchangeable with similar conclusions (Henrich 2001). For the purposes of this paper, I take CE as the more inclusive set of academic investigations looking at social transmission of behaviors, beliefs, and sentiments through a population. CE constructively focuses attention on how people use social information to make adoption decisions and how information and sentiments flow via different communication media through a society. Some choices may be learned via mass media, but others fail to transmit this way, being reliant on intimate personal interaction: a mismatch between medium and message may result in a failure of diffusion (Rogers 2003).

Grassroots First Nations activists use social transmission models to shape strategy for politically sustainable outcomes. Sustainability is usually framed with a question of the form, “If people engage in a behavior, can the environment sustain the behavior, or will the environment degrade?” CE questions the premise, asking whether people *will* engage in the behavior. A more complete notion of the sustainability of a behavior needs to include whether the behavior would diffuse into and sustain itself in a population. Even if a behavior is theoretically materially sustainable, it may not sustain itself for social reasons.

For activists, my hope is that by reframing some of the social transmission processes involved in grassroots activism, it may constructively add to the toolkit for adaptively managing environmental defense. For academics, this paper will demonstrate the utility of examining social transmission when investigating sustainability. I stick to verbal analysis; while one could mathematically formalize the verbal model presented, the benefits of increased precision are minimal, given the qualitative data used. The advantage of verbal analysis will hopefully be to make the paper more accessible.

The paper proceeds as follows. In the next section, I expand on the background of colonialism, environmental injustice, and resistance in BC. In the Theory section, I describe the theory of small vs large group organizing commonly held by grassroots activists and connecting it to cultural evolution as described in the Introduction. In the Group Bonding Theory section, I review academic theories of group bonding which parallel activist thought. In the Cases section, I review a series of fights at the intersection of First Nations sovereignty and environmental protection in BC. In the Discussion, I discuss how these cases provide support for the effective use of this theory for adaptive management of campaigns. The Conclusion summarizes the contributions of this analysis to CE as applied to sustainability analysis.

## Background

First Nations communities in BC struggle against unsustainable logging, fishing, mining, dams, and fossil fuel extraction. Resistance has escalated over the last decades, with joint mobilization around First Nations rights and environmental protection. This occurs in the context of ongoing colonial occupation, the horrors of which are rarely fully recognized outside of indigenous communities. While many of these nations were still independent countries through the early 1800 s, the mid 1800 s saw military occupation and the spread of disease, much of it spread deliberately by the Canadian government (King 2012). Over the mid-1800 s, 70–90% of the population was killed. Canada banned traditional practices and governance until the 1950s, imprisoning people for participating in core cultural practices like the gift giving potlach ceremonies (Suttles 1990). Canada established the residential school system in the late 1800s to forcibly take children from their families and violently destroy their sense of identity and culture.

Physical and sexual abuse were rampant in these residential schools. The last was only closed in 1996. Canada is only just coming to terms with its history of colonial violence (Truth and Reconciliation Commission of Canada 2015).

While much activism operates within Canadian law, there is also strong history of direct action, where activists directly obstruct projects through ‘illegal’ acts of civil disobedience, though in many cases these actions occur where the law is under contention. A primary mode of such resistance in BC is the blockade or land occupation, where First Nations groups occupy sites on their traditional territories to directly obstruct industry (Wild 1993). This contrasts nonobstructing tactics like regulated street demonstrations, media campaigns, or lawsuits. Activists often pursue multiple strategies in tandem. Occupations are simultaneous actions of environmental defense and assertion of sovereignty, often done in conjunction with lawsuits.

Many BC First Nations never signed treaties with Canada surrendering their territories. Blockades protected forests while legal cases asserting sovereignty were pursued through the Supreme Court, resulting in the 1992 Delgamukw (Persky and David Suzuki Foundation 1998) and 2014 Tsilhqot’in (Napolean 2014) rulings. These rulings, game changers for First Nations, recognize First Nations sovereignty in their traditional territories, the legitimacy of traditional hereditary government (as opposed to government imposed Band Councils, a vital point beyond the scope of this paper), and the necessity of free prior and informed consent before industrial actions on unceded First Nations territories.

Government and industry consistently attempt to circumvent these rulings. Blockades and territorial occupations are treated as illegal by the government until proven otherwise in court. They are often necessary. Industry strategically draws out cases, and if ongoing industrial destruction were not stopped ‘illegally’, then eventual legal victory would only save a wasteland (Bernard Kerrigan, Haida legal scholar, *personal communication*, August 2016). Communities successfully engaging in such direct action where I have conducted field work include Unist’ot’en, Gitksan, Tsimshian, Tahltan, and Haida communities (Frost 2016). Some other notable examples include the struggles of the Nuu-chah-nulth at Clayoquot Sound (Robinson et al. 2007), the Squamish in the Elaho Valley (Blunt 2006), the Tsilhqot’in near Williams Lake (MacCharles 2014), and the Prophet River and West Moberly First Nations against the Site C Dam in the Peace Valley. These communities are for most Canadians remote and remain “out-of-sight, out-of-mind”, underrepresented in media.

As First Nations groups are usually quick to point out, it is not a question of whether there should or should not be resource extraction; such has been ongoing for thousands of years. It is a question of sovereignty, of sustainable, balanced relationship with the more-than-human world, and of sacred relationship to place guided by traditional ecological practice.

Paralleling mobilization around First Nations sovereignty has been a rise in environmentalism in non-indigenous communities. Big early victories for the environment occurred through collaborations between First Nations and non-indigenous activists, including the protection of large tracts of traditional territory of the Haida (Haida Gwaii), Nlaka’pamux (Stein Valley), and Nuu-chah-nulth (Clayoquot Sound). Yet these early campaigns also came with cross cultural conflict and cross purposes, particularly as First Nations concerns were not universally recognized amongst non-indigenous activists.

## Grassroots activist theory and CE

Activist organizers are concerned with educating people about causes, motivating them to participate, and inculcating commitment levels sufficient to overcome resistance and endure sacrifices particular to the campaign and strategies. These are questions of social transmission, the subject of CE theory. While not framed in academic terms, activist organizers express sophisticated understandings of social transmission dynamics and their adaptive management. They discuss issues of match of medium to message, mechanisms of direct social transmission through explicit messaging and leading by example, and more indirect mechanisms of social transmission. The latter is exemplified by the bonding that arises through participation in direct action; socially transmitted action participation creates the circumstances where individuals change their beliefs and values, and thus future behavior. This is the purposeful management of cultural evolution. Many activists with whom I communicated expressed specifically a tradeoff: focus on transmitting high commitment levels through practices which diffuse slowly vs rapid transmission and higher adoption of behaviors and beliefs which generate lower commitment levels. This section describes this working model in the negotiation of cultural evolution and is a synthesis of beliefs I found to be widely held amongst direct action-oriented activists, expressed in interviews and based in many cases on personal experience.

Organizing around large groups is held to lead to shallow commitment and the threat of cooptation via large NGOs. Small-group organizing, however, is held to facilitate higher levels of solidarity amongst more tightly knit activists, more willing to engage in high sacrifice direct action and less likely to be coopted. Where campaign goals necessitate high levels of individual sacrifice, grassroots organizers express the belief that emphasizing small-tight groups is necessary to socially transmit intense commitment.

Large-scale organizing emphasizes getting information to many and actions requiring relatively lower amounts of individual sacrifice—street demonstrations, letter writing, boycotts, fundraising, etc. Large numbers of solidarity partners create economic threats through boycott and threats to politicians up for re-election. Campaigns emphasizing expansive organizing can access more donors, generating more income. These financial resources make possible a professional staff and researchers. It is very hard, however, to make a rapid jump to a large infrastructure, making collaboration with an existing NGO useful if not necessary for large-scale organizing where time is critical. Such large NGOs bring developed infrastructure, expansive social networks, and funding. This can certainly help grassroots campaigns but can also lead to cooptation as NGOs hijack social mobilization and media attention with large budgets, devoted media professionals, and established media relationships. NGOs juggle multiple priorities. They balance mission success with their own institutional survival. It is a concern that large NGOs will spend too much income on institutional support rather than on the mission. Funding is based on the appearance of success to donors, creating economic pressures to assert fast victories and take responsibility for them. Well-packaged compromises based on cosmetic success can generate such appearances of success, serving the fundraising needs of the large NGOs but failing to satisfy grassroots standards of success. Where the calamities entailed in compromise are not directly visible to non-locals, this creates a greater willingness for NGOs supported by non-locals to compromise. Describing this dynamic, I have heard many activists use the invective, “More income oriented than outcome oriented”.

By commandeering attention, NGOs divert money flows from grassroots campaigns and cause the supporting solidarity network to dissolve once cosmetic success has been achieved. Where there are conflicts in goals and lack of institutions for maintaining grassroots control, this can lead to unwanted compromise. For example, sovereignty may not be a sincere priority for urban NGOs, setting up a conflict. While there is an acknowledgement that NGOs vary in respect for indigenous grassroots leadership, conversations with grassroots activists across a variety of settings reveal general cynicism about large NGOs.

In response, many grassroots activists tightly control and minimize involvement of NGOs and cultivate smaller, tighter groups for actions. Such campaigns do well by relying on direct action tactics that can succeed with smaller groups of highly committed individuals, like occupations of transport choke points or proposed industrial sites. Such actions rely more on individual sacrifice than numbers for success.

With smaller groups, organizers can better filter for people with higher levels of commitment. When actions need to be done clandestinely, smaller groups are also less easily surveilled, and as one spends more time with each member, it is easier to know each other, decreasing the likelihood of infiltration. However, at least as important as these logistical considerations are the experienced feeling of bonding that comes with such small group organization, especially with shared sacrifice. This is the indirect social transmission of commitment. In conversations and interviews, many independently reported how engagement together in intense actions helped them bond to small activist groups and inspired adherence to sacred values, engendering greater levels of willingness to sacrifice for shared cause. These intense bonding activities can take many forms, from a confrontational action, like a pipeline blockade or building lock down, to sacrifice of time and effort in the construction projects such as at Unist’ot’en Camp and other occupations. Through greater willingness to sacrifice, more resistance to compromise, and strong adherence to group values (and thus First Nations objectives), such small-intense groups can potentially hold out longer against industry/government in more high-pressure circumstances. While investing in social transmission of direct action participation is perhaps slower and results in lower numbers of ‘adopters’, those who adopt are both filtered for higher commitment and induced into higher commitment through participation.

Actual organizing is more complex than a simple dichotomy, often involving mixed strategies. Small and large group organizing are not necessarily opposed. Work defending First Nations traditional territories often involves small groups of intensely devoted activists aided by extensive supporter networks, in managed coalition with NGOs. Small groups can be formed in large-scale actions. Large-scale actions can use the space and time provided by small-scale direct action to potentially mobilize large numbers for change at the ballot box. Large support networks can help maintain visibility for front-line actions, pressuring police to act within the rule of law and reducing the risk of violence against front-line activists.

Under what circumstances may a strategy succeed? Time pressure, levels of sacrifice, and choke points are considerations. Where industrial action can be achieved quickly, it is often necessary to engage in direct action to prevent irreversible destruction. In such instances, legal cases alone are inadequate. Legal ambiguity allows for large-scale destruction to proceed during the slow, strategically drawn-out process of court proceedings. Once a company builds an oil pipeline, it is far harder to fight the flow of oil. In these spaces where street protests are too slow to mobilize or have effect, ‘illegal’, high risk, confrontational action is often required.

‘Choke points’, where industrial processes pass through a small area, create opportunities where small groups of actors can have large effects. If a forest is only accessible through one logging road; obstructing such a road could block a logging operation. Pipelines going through mountains only have a finite number of possible routes. If there are choke points, then small numbers of devoted actors can have powerful effects. If solidarity commitments are shallow, industry/government erodes resistance by increasing individual consequences (increased legal penalties, violence, etc.). Where government can inflict severe individual consequences, in such cases commitment is more important than numbers.

While CE and ‘social learning’ are not terms bandied about amongst activists generally, in attending to recruitment and how action participants acquire commitment, grassroots organizers attend to social transmission dynamics in adaptively managing campaigns: these are implicit theories of cultural evolution. Cultivation of solidarity and commitment to values both are themselves socially transmitted and result from recruitment to specific (socially transmitted) actions. Framed in terms of CE, participation in high-sacrifice direct action diffuses slowly for a given investment in transmission, limited by the intimacy necessary for transmission. It, however, both directly transmits high commitment and sets up the circumstances where people acquire yet higher commitment. In contrast, large-scale campaigns rely on fast, extensive diffusion of values and elicit lower commitment. While large numbers can facilitate powerful actions, lower individual commitment limits what actions can be successfully pursued. Grassroots campaigns exhibit adaptive management of these processes through ongoing attention to campaign needs and support, shifting investment in large- or small-scale aspects of campaigns.

## Group bonding theories and the social transmission of values

Activist theory of small vs large group organizing closely parallels three bodies of social-science research: the works of Ostrom, Whitehouse, and Atran. While these theories do not reference cultural evolution, they all describe dynamics of social transmission and collective maintenance of values and behaviors.

### Ostrom: socio-ecological systems (SES)

Ostrom’s SES framework identifies design principles that facilitate the evolution of institutions which successfully manage common pool resource problems (Ostrom 1990). These principles include clearly defined, self-determined, autonomous groups of limited size with collective choice arrangements, and institutions of mutual monitoring. This proposal is supported by extensive empirical study. Such groups display the ability to adaptively manage local commons. Practically, monitoring a small, closed group for compliance to cooperative norms is easier, and collective decision making becomes more challenging as group size increases. It is easier to develop deeper trust in norms of cooperative altruism within the circle of people whose faces you recognize than within groups too large to afford personal acquaintance, a principle of limits on group size arrived at independently elsewhere (Dunbar 1993). Ostrom’s work was developed in the context of the evolution of institutions coping with environmental commons, where the benefits of personal sacrifice are experienced by a group. The political solidarity needed in direct action campaigns is also a common problem: one sacrifices in political struggle for the benefit of others. While the norms of grassroots activists involve sacrifice for the benefit of far larger groups than the circle of direct action co-participants, there are many sacrifices where the benefits are largely held in common with fellow action participants, i.e., solidarity in the face of police threats and literal prisoner’s dilemmas. Grassroots activists and the SES community both recognize how the practicalities and personal connection of small groups facilitate reproduction and transmission of shared norms.

### Whitehouse: dual modes of religiosity (DMR)

Shared physical practice facilitates identity formation and in-group altruism in a wide variety of contexts (McNeill 1995). DMR was developed to explain the cognitive underpinnings of *divergent* forms of bonding amongst religions, associated with different society scales (Whitehouse 2004). DMR proposes two primary ritual modes. *Imagistic* rituals are highly intense, performed infrequently, and are associated with small, tightly bonded identity groups. The Sundance ritual, involving fasting, endurance dancing, and ritual piercing, would be an iconic example. Such practices engage ‘flash bulb’ memory, creating strong associations with co-participants. *Doctrinal* rituals are low intensity, performed frequently and are associated with larger societies and lighter bonds. Bowing to Mecca five times per day and Catholic Mass would be iconic examples. These practices bond an individual to an abstract sense of identity based around shared norms, rather than to specific individuals. While they create lower intensity bonds than imagistic mode rituals, they can bond together much larger identity groups. This association between mode of practice and scale and intensity of social organization has been well established through examination of ethnographic records (Atkinson and Whitehouse 2011). Societies can mix practices for different contexts. For example, militaries use practices like close order drills (boring, repetitive), to bond the mass of the military into an identity group, but will engage in excruciating training exercises in boot camp (infrequent, high intensity) to bond smaller units into groups willing to impulsively die for each other. The effects of socially transmitted imagistic vs doctrinal mode practices on group identity directly parallel the experiences that activists report with small- vs large-scale organizing in transmitting values.

### Atran: psychology of the sacred

Through extensive, collaborative research on the psychological underpinnings of support for extremism (Atran 2010), Atran identified two modes of values: a mundane, rational mode and a *sacred* mode. When dealing with mundane values, people willingly make trade-offs, compromise, and (more or less) rationally update their beliefs based on evidence. However, under the combination of conditions where a rhetorical position is a marker of identity, group identity is held strongly, *and* there is a perception of ‘group threat’, people react emotionally to offers of negotiation or to information that threatens the truth value of the rhetoric as a ‘group threat’. Experiencing group threat, individuals amplify group markers—in this case, values. Emotional provocation is hard to avoid around sacred group markers, and when thus agitated, the individual will counter-intuitively react with an *amplification* of values or belief rather than with compromise or incorporation of new information. This parallels earlier anthropological findings (Rappaport 1999). Key to the transmission of the kind of exceptionally strong sacred values that lead to extremism and radical self-sacrifice is shared identity formed around shared experiences in small groups. Extremism emerges out of a runaway version of familiar acts of social bonding in small groups combined with a sense of external threat. By Atran’s theory and extensive documentation, terrorist networks are less well described as hierarchical, centralized organizations bonded around a doctrine than as loosely and flexibly connected clusters that are self-sustaining, self-motivating, and self-radicalizing around sacred values. The intensity of their commitment hinges critically on the intensity of their connection to each other. This parallels activists’ observations about small group organizing and also research on the 1964 Freedom Summer anti-racism mobilization which found that close ties were the strongest predictor of participation (McAdam 1986). ‘Sacred’ refers not necessarily to spirituality or religion, but more broadly to such *non*-*compromise* based around shared group identity.

Atran’s study was in the context of extremist violence. However, these psycho-social dynamics are more general, and ‘radicalization’ is not limited to radical support for violence. Shared experiences of direct action civil disobedience in small groups create shared, felt identity. The formation of sacred values and in-group/out-group dynamics described by Atran are also familiar dynamics in radical activist contexts. Atran’s work emphasizes how such sacred values lead to noncompromising negotiation. In some contexts, this is not necessarily a bad thing. The sustainability of some outcomes may rely pivotally on the social transmission of resistance to compromise, when a moment of wavering may lead to effectively irreversible environmental consequences.

## Cases

The Clayoquot Sound demonstrations of the early 1990s are a primary early reference for fights at the intersection of First Nations sovereignty and environmental defense (Robinson et al. 2007). Initiated by the Nuu-chah-Nulth to protect their traditional territories from clear-cut logging on Vancouver Island, the protests attracted an extensive network of mostly white environmental activists to blockade logging roads. This resulted in the largest mass arrest for an environmental protest in North American history, with over 850 arrested. Clayoquot is primarily portrayed as a success for having protected a significant amount of the remaining watershed and their intact old growth forests. However, many grassroots activists have expressed how they felt that their concerns were sidelined as the urban activists, with their expansive support networks, swept in, taking over protests and negotiations. Many First Nations activists with whom I talked, referring to such activists as ‘white knights’, report experiencing this as an extension of racist colonialism, as indigenous leadership is sidelined. They argue that NGOs, arriving late in the game, took over negotiations, cut compromises, pitched the results as success to media, and leveraged this with funders for more organizational funding. Even though the entire Sound was given an official designation as a ‘biosphere reserve’, logging continued in much of Clayoquot, and not all intact old growth was formally protected. While the logging that continued was ‘First Nation owned’, those in charge have been pushing to log intact old growth areas in order to service debts incurred in buying out timber licenses (Bunsha 2013). First Nations having to buy out timber licenses on their own territory rather than simply having their sovereignty affirmed are part of the legacy of compromise that has left a bitter memory amongst grassroots activists negotiating relationship to NGOs.

A contrasting example is the logging blockade campaign in the Elaho Valley (Squamish territory). Instead of large NGO support, activists relied on small-group organizing and direct action, including tree sits and other forms of obstruction. While the Clayoquot protests resulted in arrests, the kind of treatment received was different in the less populated Elaho, with more severe violence against activists and severe legal repercussions (Blunt 2006). However, the smaller, highly devoted group persisted and achieved better results than if a similar compromise had been settled for. Certainly, there were many contextual differences, but the grassroots organizers felt clear that shallowly committed large groups would not have been able to save the forests given the intensity of physical confrontation from industry and police. Because of pressures from direct action, the Squamish was able to achieve unique protection to the area under the control of traditional hereditary leadership (Mitchell 2007).

One of the latest large-scale campaigns has been the Great Bear Rainforest Initiative to protect the temperate rainforest of the central coast of BC, spanning the traditional territories of many First Nations (Macleod 2016). Formal negotiations were forced via a boycott of BC timber, organized by a coalition of large NGOs collaborating with local activists. The economic impacts of the international boycott were key to bringing industry to the table. However, the NGOs were criticized by some grassroots activists for agreeing to compromises and for excluding grassroots organizations from negotiations (Stainsby and Oja Jay 2015). The Nuxalk, being unwilling to compromise, was excluded from the negotiations. Enormous tracts of territory are nominally protected under the agreement (McAllister 2016). The allowances within the regulations, however, make the protections seem inadequate in limiting logging in the Great Bear Rainforest in the eyes of many scientists and local activists, upset about the impacts of ongoing clear-cut logging. While permitted logging has limits on cut size and boundary zones around streams, these are not well enforced, as can be seen via a ferry ride along the coast. The NGOs signed unusual agreements to not publicly criticize the end results. With the resulting lack of NGO criticism, the publicly perceived success of the initiative makes struggles to resist ongoing logging in sensitive areas difficult.

The Unist’ot’en Camp land occupation is the most well known of the current wave of First Nations grassroots campaigns (Frost 2016). Their traditional territory occupies a bottleneck in a rugged mountainous landscape. It is difficult for a pipeline from the tar sands oil or fracked gas operations of Alberta to reach the north coast without going through their territory. A small group of activists have prevented pipeline construction, backed by a large network of supporters. They have replicated aspects of previous logging blockades but also learned lessons from these about the dangers of NGO involvement. The Unist’ot’en (a Wet’suet’en clan) maintains strict control over how people interact with the campaign, including vetting of volunteers and requirements of all visitors to go through traditional protocol rituals that acknowledge First Nations leadership (Frost 2015). The Unist’ot’en campaign has many sides, but is centered around a network of activists devoted to living year-round on the land, revitalizing Unist’ot’en traditional lifeways and safeguarding the territories against industrial incursions in confrontations that often involve the police. With help from non-indigenous supporters, the Unist’ot’en has built a year-round habitable cultural center directly on critical pipeline routes in the middle of their traditional hunting grounds in the harsh mountain environment of northern BC. Keeping up check points during times of high intensity confrontations with industry and police requires large sacrifices of time and can be quite stressful. Yet the pressures of burn out seem to be counterbalanced by the sense of solidarity developed through participation. The work of this smaller network of highly devoted activists has given time and space for a large network of supporters to develop and for other groups who would similarly be impacted by the pipelines to pursue law suits that will likely eventually block them, as occurred already with the Enbridge Northern Gateway Pipeline (Proctor 2016). They have explicitly rejected working closely with large NGOs, relying instead on a less institutional, but strongly committed network of supporters.

A particularly impactful tool of the Unist’ot’en has been hosting gatherings combining volunteer work, direct action training, and activist networking. The first annual Action Camp was organized in 2010 so the Unist’ot’en themselves could learn from other experienced activists, including previous blockaders in other territories. These yearly gatherings serve many functions: the horizontal transmission of skills and techniques for activism, the active recruitment of new supporters for Camp, and bonding of activists through shared investing of time and energy in construction projects. Action Camp itself is a local adaptation of the practice in the wider activist community of gatherings for horizontal information/skill sharing. Activists come together to share lessons from their experiences, allowing the accumulation and synthesis of useful information.

Learning from each other’s successes and failures facilitates the constructive evolution of practice.

Participating in construction work builds community, motivating people to return, contribute further, and bring friends. As activists stay to participate in the ongoing year-round occupation in sometimes tense interactions with pipeline workers, sense of commitment intensifies. Many with whom I have talked expressed how the shared experience of volunteering served to increase their sense of commitment to shared group values of environmental protection and de-colonization.

The pattern of First Nations land occupations asserting sovereignty continues to be replicated across northern BC, with each subsequent manifestation picking up on lessons from past actions. The Unist’ot’en Camp land occupation has served as a model for other successful occupations, like the Lelu Island occupation by Gitwilgyoots (Tsimshian) and the Luutkudziiwus (Gitksan) occupation at Madii Lii. In these cases, there is a similar dynamic where a geographic bottleneck means that a specific site can be occupied by a smaller group of devoted activists, engaging in community-focused construction projects on proposed industrial sites. As Madii Lii is quite close to Gitksan communities in the Hazleton, they have been able to strongly emphasize use of the blockade camp for cultural revival as they successfully assert control over hunting in their territories while also preventing pipeline work. The Lelu Island occupation particularly mirrors the Unist’ot’en case, where a bottleneck allows a small group to fend off an enormous industrial project. Pursuing direct action occupation with small numbers, the Lelu Island action successfully delayed the $30 billion Petronas LNG export facility to the point where Petronas gave up on the project (Frost 2017b). Both campaigns have also declined to work closely with large NGOs, working instead with small, local NGOs, like the Skeena Watershed Conservation Coalition and Skeena Wild, with more personal connection and willingness to uphold indigenous leadership.

The campaigns learn from each other strategies for adaptive management of recruitment and commitment. They balance investment in small-group and large-group organizing to maintain sufficient levels of recruitment to the small circle of highly committed activists at camp and to the wider networks necessary to support the campaigns. They also invest in practices of information exchange to accelerate social learning amongst campaigns. By strictly regulating and sometimes rejecting involvement of NGOs, they maintain local control and resist compromise.

## Discussion

CE is the theory of changes of behavior through social transmission. Other models that do not focus on how information, behavior, and sentiment are socially transmitted miss critical details of the unfolding of grass roots activism. While an individual decision model may capture how individuals make decisions based on their values, a CE approach emphasizes how people acquire those values. The adaptive management of campaigns illustrates how attention to values transmission and recruitment is central to mission success. In cases like those of Unist’ot’en Camp and Lelu Island, industry work crews were ready and waiting for years to carry out work. It was the ongoing and determined resistance of front-line activists that held off construction and avoided irreversible destruction. This commitment was cultivated using strategies of campaign organizing learned over a history of blockades and occupations and paralleling several threads of social science research. As with other blockades in Haida, Tahltan, Gitksan, and Squamish territory, waiting for legally sanctioned resistance to run its course would have been environmentally devastating. Reliance solely on large-scale organizing would have thus been unlikely to provide the intensity of emotional commitment necessary for direct action. While Clayoquot demonstrated how large groups could be motivated to offer sacrifice, the remoteness in many of these other cases and the levels of sacrifice necessary make such mobilization less plausible. It is a different level of sacrifice to risk a week in jail with hundreds of comrades and small fines than physical violence and years in jail on felony charges, as is sometimes at risk in more isolated areas.

There are many variables in a campaign that shape how much sacrifice is required of individuals for success. A bottleneck may create a situation where a small group willing to engage in high sacrifice can hold off an enormous industrial project long enough for legal processes or large-scale political organizing to enact longer term change. This is illustrated by the Unist’ot’en, Lelu, and Madii Lii cases. A campaign closer to a more privileged white urban community may be able to concentrate more on rapid large-scale organizing tactics, as less severe consequences are likely to be applied to them for a given level of resistance. In a case where a specific industrial action may be carried out rapidly with irreversible consequences, small group direct action tactics may be essential. It is also certainly the case that shifts in the energy economy have played into the hands of grassroots activists. In the case of Lelu, a primary justification given by Petronas for withdrawal was lower prices of natural gas. Direct action bought time for the changing economy to stop the project.

The legal circumstances of non-treaty First Nations in Canada are an important consideration. The Delgamukw and Tsilhqot’in rulings established strong rights for non-treaty nations. These nations were also some of the last to be colonially dominated in North America. In many cases, their cultures have been maintained remarkably well and their formal government persisted with continuity. The Canadian government still allows industrial actions in violation of these rulings, forcing Nations to go to court to defend their rights. The fact that these rulings exist, though, means that direct action has greater perceived legitimacy and potentially can achieve positive results in shorter spaces of time when done in parallel with law suits. Direct action tactics could play out differently in places with less rule of law or legal rights.

Small-group organizing has the drawback that the scope of individual campaign networking is small, limiting the transfer of tactics. Practices like Action Camp have developed over the years addressing this problem, to support recruitment and information transfer, accelerating the positive evolution of direct action strategies and the spread of their use.

For non-indigenous solidarity partners, it is important to see how the relative speed of transmission of messages that urban environmental networks access can be a two-edged sword. Urban, non-indigenous activists can numerically overwhelm an action with rapid recruitment via urban-centered media. Non-indigenous activists wanting to challenge colonial legacies need to act collaboratively rather than in a way that perpetuates racially structured power. The rise and ubiquity of First Nations protocol practices are a response to this, helping shift the culture of nonindigenous urban environmentalists to respect First Nations rights and leadership. It is now de rigor to begin any sort of progressive public gathering in BC with protocol, acknowledging that the meeting itself occurs on unceded First Nations territory.

In these cases, solidarity between First Nations and non-indigenous environmental activists has led to successful outcomes for both sides, with some growing pains with regards how to work together. A fear sometimes expressed in environmental circles about investing everything in such collaborations regards what happens when there is environmental conflict that does not have a First Nations group coming forward to defend the land with a clear territorial claim. A current example is Digby Island (Frost 2017a). Digby parallels Lelu in most ways—an Asian petrochemical company (Nexxen) proposing an LNG facility at the mouth of the Skeena River, threatening critical salmon habitat. However, to date no one has stepped forward to identify themselves as having traditional claim to the area, with the hereditary titles to this area being less clearly maintained than with Lelu, perhaps due to more severe colonial impact on the hereditary government. The local non-indigenous activists fighting to protect the land and waters there have not had the success that the traditional hereditaries have had with rallying support for Lelu.

While large urban NGOs have been criticized for perceived sidelining of First Nations, small local NGOs like the SWCC have supported First Nations leadership. The big NGOs also to varying degrees are adapting to the shifting landscape of sentiment and law with more explicit support for First Nations leadership. The Great Bear Rainforest Initiative, while critiqued, critically involved First Nations voices in negotiations. Some, like the David Suzuki Foundation, have been quite active in supporting First Nations voices and are well received for that. It is likely that as grassroots First Nations activists achieve more successes that large NGOs will continue to strategically adapt to support First Nations.

Social transmission of grassroots strategies is not simply blind replication. Details are learned from different sources and local context requires innovation and ongoing experimentation. The Madii Lii camp of the Luutkudziiwus followed and was both inspired by and able to learn from the Unist’ot’en Camp occupation. However, the circumstances were different, both geographically and socially, with Madii Lii much closer and easier to access for the clan. It is easier then for Madii Lii to emphasize use of the camp for cultural practices of the clan and de-emphasize large gatherings of non-indigenous solidarity partners. This ongoing experimentation with alternatives allows for practical ongoing research on decolonizing strategies as well as solutions that better fit local circumstances.

Reality is always more complex than our simple models. The range of commitment displayed by large groups includes impressive displays of self-sacrifice. The masses willing to be arrested at Clayoquot were impressive. Likewise, at Unist’ot’en Camp, hundreds of people have over the years spent weeks or months at a time at the camp volunteering long hours of construction work. The levels of individual commitment that large groups can mobilize are not insignificant. There is also both a mix of strategies within campaigns and a blurring of levels. Large group aspects of campaigns, like Action Camp, serve as recruiting grounds for those willing to engage in more intense direct action. While differences in the experiences and effects of these kinds of actions should be recognized, we also should not hold them as completely distinct processes.

While some view the Clayoquot Sound and Great Bear Rainforest outcomes uncritically as successes and most would agree that there were positive outcomes relative to the status quo, some grassroots activists and traditional hereditary leaders see failures that could have been avoided. With Clayoquot, the necessity for buying timber licenses on their own traditional territories is an example of a compromise that some feel could have been avoided. With the Great Bear Rainforest, it is too new to tell exactly how it will unfold, but some problems have already been pointed to and excluded actors feel that their views were not represented. On the other hand, actions like Unist’ot’en Camp, Lelu Island, and Madii Lii have thus far avoided compromises that were entailed in both Clayoquot and the Great Bear Rainforest campaigns.

There has been a steep learning curve for grassroots activists in navigating relationship with NGOs. With attention to maintenance of First Nations leadership, NGOs can be very helpful in generating support networks. The positive outcomes in the cases of the Madii Lii and Lelu cases were in part facilitated by the help of local NGOs committed to First Nations leadership and agency. Unist’ot’en Camp’s less formal network of supporters has been likewise enormously helpful. Large-scale organizing can be used to fundraise and gain political support in far-flung networks. The financial support can also convert high sacrifice situations into lower ones. Unist’ot’en Camp, with financial support from a large network of donors, built winterized buildings to house activists comfortably year-round, lessening their sacrifice to manageable levels. In the direct action campaigns, visibility maintained by a large network of supporters helps keep industry and police behavior in check. Support from larger groups can shift away some part of the burden faced by front-line activists.

## Conclusion

Several threads of behavioral studies highlight the increased ability of small-group organizing to cultivate tight bonds that facilitate high levels of altruism and adherence to group values. Observation in the context of First Nations grassroots struggles in northern BC replicates these findings. The practice-based theory of small- vs large-scale organizing examined in this paper highlights the increased efficacy of working in small groups in contexts of direct action resistance requiring large individual sacrifices. First Nations led grassroots organizations negotiate the social transmission of values, recruitment, and strategies of resistance amongst causes, adaptively attending to the details of these social learning processes as campaigns unfold. This is adaptive management of CE. Using small-scale organizing of direct action campaigns, they have been able to successfully transmit amongst activists sufficiently high levels of solidarity to achieve group objectives in often quite stressful circumstances requiring large sacrifice. By relying less centrally on large-scale organizing via urban centered NGOs, they have been able to maintain local control of the campaigns, making unnecessary compromise less likely. In the case of remote communities with less resources, direct action is often necessary to prevent irreversible environmental damage that legally sanctioned tactics would be incapable of stopping. Matching medium to message, the necessity for high commitment suggests, then, small-scale organizing.

Valuable lessons have successfully been passed on from campaign to campaign. Institutions of transmission have developed in these communities, increasing the ability to copy and modify successful strategies from context to context. Grassroots activists not only adaptively manage social transmission of participation and commitment, but manage the process of strategy evolution itself via strategy exchange institutions like Action Camp. Attention to social transmission dynamics (CE) is leading to remarkable successes in fights for environmentally and politically sustainable outcomes.

CE does not supply a cookie cutter solution, simple conclusions like “small-scale organizing is better”. Rather, CE reveals elements that need attention as campaigns unfold. In this case, it is attention to the transmission of not just information, but of commitment, requiring a matching of medium to message. One applies CE by tracking dynamics of social transmission, using this information to reshape investment in different levels of organization continuously. Adaptive management entails ongoing attending to recruitment, commitment levels required by current actions, and innovation and information flow amongst campaigns. Without a rich, practice-informed theory of social transmission dynamics (CE) of action participation, activists would not be able to successfully manage campaigns.

Future research could constructively focus on the synthesis of small- and large-scale organizing.

While Whitehouse’s DMR posits that highly arousing practices bond small groups, cases like Clayoquot demonstrate that large groups have been inspired to significant levels of self-sacrifice through shared experiences and values. Several research questions arise. What are the differences in longevity, stability and intensity of solidarity that arise from similar amounts of sacrifice in larger vs more intimate actions? How are large-scale events recruiting grounds for intense small-scale actions? What institutional patterns have developed for synthesizing small- and large-group organizing, and are these patterns replicated across campaigns? What are the various ways that large-scale solidarity networks shift burden away from front-line defenders? Also, while there is a common belief amongst grassroots activists that large NGOs have conflicts of interest as they juggle mission success and institutional survival and expansion, it would be a constructive future line of research to see to what extent this affects mission success, what variance there is amongst NGOs, and how this variance affects the survival, rise, and fall of NGOs.

The research of Ostrom, Atran, and Whitehouse would come as no surprise to many grassroots activists. While direct action campaigns are pursued for functional goals of stopping destructive industrial action and there is clear understanding that solidarity is essential, by highlighting the function of the actions themselves in developing commitment to values, activists may be able to fine tune these actions to best facilitate solidarity.

This paper contributes to the field of cultural evolution by presenting how processes of social transmission are self-consciously adaptively managed. It contributes to sustainability literature by demonstrating how processes of social transmission are in some cases vital for environmentally sustainable outcomes.

## References

[CR1] Atkinson QD, Whitehouse H (2011). The cultural morphospace of ritual form: examining modes of religiosity cross-culturally. Evol Hum Behav.

[CR2] Atran S (2010). Talking to the enemy.

[CR4] Blunt Z (2006) Shaking the tree: an eco-defender’s ordeal. http://www.zoeblunt.ca/2009/11/19/shaking-the-tree-an-eco-defenders-ordeal/

[CR5] Boyd R, Richerson PJ (1985). Culture and the Evolutionary Process.

[CR6] Bunsha D (2013) What clayoquot sound faces now: a historic opportunity to protect BC old growth forest, through new partnerships. The Tyee, Columbia, pp 1–6. http://thetyee.ca/Opinion/2013/08/19/Clayoquot-Faces-Now/

[CR7] Dunbar RIM (1993). Coevolution of neocortical size, group size, and language in humans. Behav Brain Sci.

[CR8] Frost K (2015) Protocol on First Nations Territory. http://www.weeatfish.org/protocol-on-first-nations-territory/

[CR9] Frost K (2016) We Eat Fish (a film, travel blog, and web-based documentary on the interlocking issues of First Nations sovereignty, fights against oil and gas development, and cross-cultural solidarity building in BC, Canada). Canada. http://www.weeatfish.org

[CR10] Frost K (2017a) Digby Island proposed Nexen Aurora LNG (fracked gas) export facility, BC, Canada. Environmental Justice Atlas. http://ejatlas.org/conflict/digby-islandproposed-nexxen-aurora-lng-fracked-gas-export-facility-bc-canada

[CR11] Frost K (2017b) Gitwilgyoots and Lelu Island vs proposed Petronas LNG export facility, Canada. Environmental Justice Atlas. https://ejatlas.org/conflict/gitwilgyoots-and-leluisland-lax-uula-vs-proposed-petronas-lng-export-facility

[CR12] Henrich J (2001). Cultural transmission and the diffusion of innovations: adoption dynamics indicate that biased cultural transmission is the predominate force in behavioral. Change.

[CR13] King T (2012). An inconvenient Indian.

[CR14] MacCharles T (2014). Supreme Court grants land title to B.C. First Nation in landmark case.

[CR15] Macleod A (2016). “Solutions Are Possible”: Great Bear Rainforest Land—use deal reached.

[CR16] McAdam D (1986). Recruitment to high risk activist: the case of freedom summer. Am J Sociol.

[CR17] McAllister I (2016) UNFILTERED: The Great Bear Rainforest Agreement. https://pacificwild.org/news-and-resources/great-bear-blog/unfiltered-the-great-bearrainforest-agreement

[CR18] McNeill WH (1995). Keeping together in time: dance and drill in human history.

[CR19] Mitchell A (2007). Province, Squamish Nation reach land use agreement.

[CR20] Napolean V (2014) Tsilhqot’n Law of Consent. pp 1–18

[CR21] Ostrom E (1990). Governing the commons: the Evolution of Institutions for Collective action.

[CR22] Persky S, Foundation David Suzuki (1998). Delgamuukw: the supreme court of Canada decision on aboriginal title.

[CR23] Proctor J (2016) Northern Gateway pipeline approval overturned. CBC News

[CR24] Rappaport R (1999). Ritual and religion in the making of humanity.

[CR25] Robinson JL, Tindall DB, Seldat E, Pechlaner G (2007). Support for first nations’ land claims amongst members of the wilderness preservation movement: the potential for an environmental justice movement in British Columbia. Local Environ.

[CR26] Rogers E (2003). Diffusion of innovations.

[CR27] Stainsby M, Oja Jay D (2015) Offsetting resistance: the effects of foundation funding and corporate fronts from the Great Bear Rainforest to the Athabasca River, vol 1

[CR28] Suttles W (1990). Handbook of North American Indians.

[CR29] Truth and Reconciliation Commission of Canada (2015) Honouring the truth, reconciling for the future Summary of the final report of the Truth and Reconciliation Commission of Canada. Winnipeg

[CR30] Turner NJ, Ignace M, Ignace R (2000). traditional ecological knowledge and wisdom of Aboriginal Peoples in British Columbia. Ecol Appl.

[CR31] Whitehouse H (2004). Modes of religiosity: a cognitive theory of religious transmission.

[CR3] Wild N. Blockade (1993) Canada. http://www.canadawildproductions.com/film/blockade/

